# Sinonasal Metastasis of a Pancreatic Mucinous Adenocarcinoma: An Unusual Case Report

**DOI:** 10.7759/cureus.91649

**Published:** 2025-09-05

**Authors:** Valerie Chatzopoulos, Yolene Lefebvre, Riccardo De Angelis, Nicolas De Saint-Aubain

**Affiliations:** 1 Department of Radiology, Hôpital Universitaire de Bruxelles (H.U.B), Brussels, BEL; 2 Department of Pathology, Institut Jules Bordet, Brussels, BEL

**Keywords:** ethmoidal sinus lesion, gastrointestinal cancer metastasis, pancreatic adenocarcinoma, paranasal sinus tumor, sinonasal metastasis

## Abstract

Metastases to the sinonasal tract are exceedingly rare and often signify advanced-stage disease. Among known primary sites, pancreatic adenocarcinoma is an exceptional origin for sinonasal dissemination.

We report the case of a 66-year-old male previously diagnosed with mucinous pancreatic adenocarcinoma, who presented with persistent right-sided nasal obstruction. Imaging revealed an aggressive ethmoidal mass extending into the orbit, nasal cavity, and anterior cranial fossa. Histopathological analysis following biopsy proved it was consistent with pancreatic origin. Given the patient’s known cancer history and the radiological and immunophenotypic findings, a diagnosis of sinonasal metastasis from pancreatic adenocarcinoma was confirmed.

This case underscores the diagnostic challenges presented by sinonasal masses in cancer patients, especially when symptoms are nonspecific. It highlights the rarity of sinonasal metastases from pancreatic cancer and the importance of including metastatic disease in the differential diagnosis of sinus masses, especially in oncologic patients.

## Introduction

Sinonasal metastases represent an exceedingly rare clinical finding, accounting for less than 1% of all sinonasal tumors [1]. The most frequently reported primary sites include the kidneys (especially renal cell carcinoma), breast, lung, and thyroid [2]. Metastases from the pancreas are exceptionally uncommon [3]. This rarity is attributed to the anatomical isolation and relatively low vascularity of the sinonasal tract, which typically limits hematogenous dissemination [4].

Symptoms of sinonasal metastases are often nonspecific and may mimic benign or inflammatory sinus disease, such as nasal obstruction, epistaxis, facial pain, or swelling [2,5,6]. As a result, diagnosis is frequently delayed or incidental. Imaging studies may suggest an aggressive lesion but lack specificity regarding the lesion’s origin. Hence, histopathological evaluation with immunohistochemistry is essential for definitive diagnosis [7].

The case presented here describes a mucinous pancreatic adenocarcinoma metastasizing to the ethmoid sinus, a site rarely reported in the literature [3]. Mucinous pancreatic adenocarcinomas are rare, representing less than 2% of pancreatic cancers, and are characterized by abundant extracellular mucin pools with floating tumor cells [8]. This case highlights not only the diagnostic complexity of sinonasal metastases but also the importance of considering metastatic spread in patients with atypical sinonasal symptoms and a known malignancy. It also shows the essential role of histopathology and immunohistochemistry in distinguishing primary from metastatic disease and reinforces the need for heightened clinical suspicion in oncologic patients with new ENT symptoms.

## Case presentation

A 66-year-old male with a medical history of mucinous adenocarcinoma of the pancreas, diagnosed in December 2023, was referred to the ENT emergency department in March 2025 for evaluation of persistent right-sided nasal obstruction. He denied associated epistaxis, rhinorrhea, fever, or visual changes. His previous history of oncologic management included two cycles of chemotherapy (gemcitabine-based) and radiotherapy, with stable systemic disease. Two surgical attempts at curative resection (Whipple procedure) were aborted due to vascular involvement. The tumor encased the common hepatic artery (>180°) and was in close contact with the superior mesenteric artery, making dissection unsafe.

**Figure 1:**
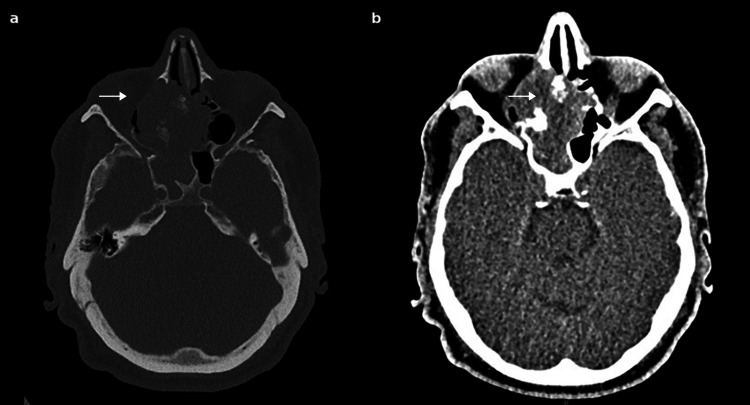
Axial CT scan findings Axial CT scan of the sinonasal cavities in bone window (a) and soft tissue window (b) showing an expansile mass filling the right ethmoidal sinus with extension into adjacent structures (arrows).

**Figure 2:**
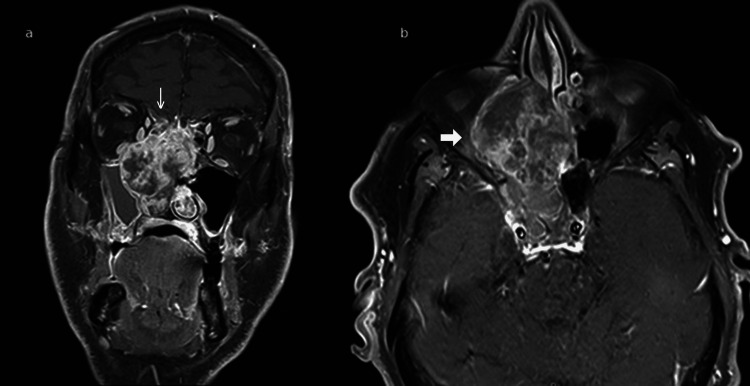
Post-contrast T1-weighted with fat saturation MRI findings (a) coronal view showing an infiltrative mass extending into the left orbit and skull base (thin arrow); (b) axial view demonstrating the same lesion invading adjacent bony and soft tissue structures (thick arrow).

On physical examination, there was tenderness over the right maxillary and frontal sinuses, but no visible masses were noted on anterior rhinoscopy. Nasal endoscopy revealed a large mass occupying the right nasal cavity. A non-contrast CT scan of the sinonasal cavities demonstrated complete opacification of the right maxillary, ethmoid, and frontal sinuses with evidence of bone erosion (Figure 1). MRI confirmed a heterogeneous enhancing mass involving the ethmoid sinus, the lamina papyracea, and the medial orbital, in contact with the inferior rectus and medial rectus muscles, extending into the anterior cranial fossa, though no overt meningeal involvement was observed. Infiltration of the right maxillary sinus with secondary fluid obstruction was observed too (Figure 2). No cervical lymph nodes were observed. This lesion, considered a primitive tumor, was classified as cT4N0.

**Figure 3:**
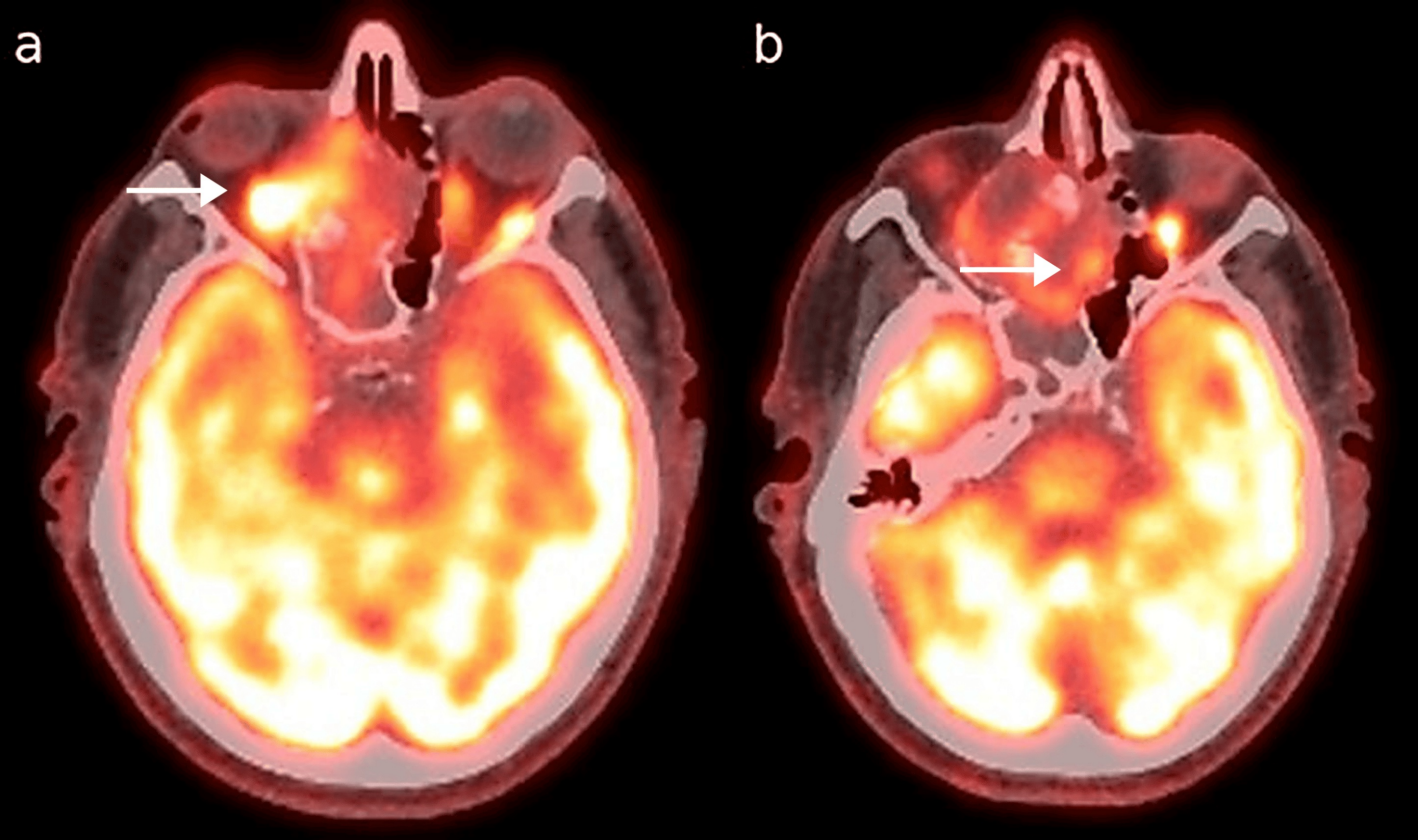
Axial fused PET-CT findings Intense fluorodeoxyglucose (FDG) uptake in the right nasal fossa/ethmoidal cavities and peri-orbital regions (white arrows), consistent with hypermetabolic tumor invasion. a and b illustrate different axial levels.

**Figure 4:**
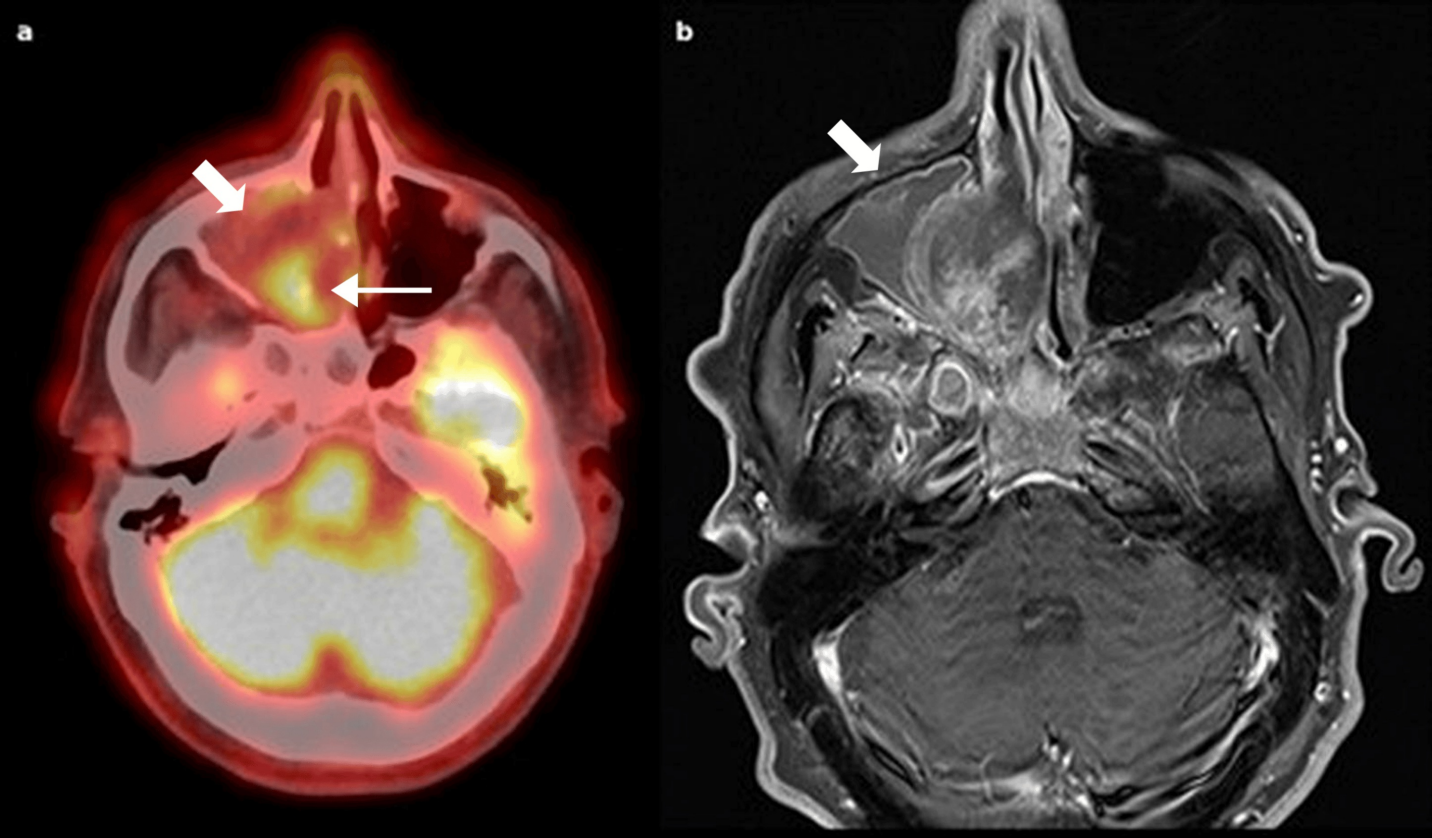
Axial fused PET-CT and corresponding contrast-enhanced T1-weighted MRI Axial fused PET-CT image (a) and corresponding contrast-enhanced T1-weighted MRI (b) at the same anatomical level. Note the absence of fluorodeoxyglucose (FDG) uptake in the right maxillary sinus, consistent with fluid content (thick arrow). Thin arrow representing the FDG uptake into the inferior ethmoidal part of the mass.

A whole-body fluorodeoxyglucose (FDG) PET/CT was performed on April 3, 2025. It revealed moderate hypermetabolic activity in the right nasal fossa, with extension into the ethmoidal sinus and involvement of the right orbital floor (Figure 3). The right maxillary sinus was completely opacified but showed no metabolic activity (Figure 4). Extra-cephalic findings included multiple hypermetabolic foci in the perihilar hepatic region and subhepatic area, suggestive of either malignant recurrence or inflammatory changes.

**Figure 5:**
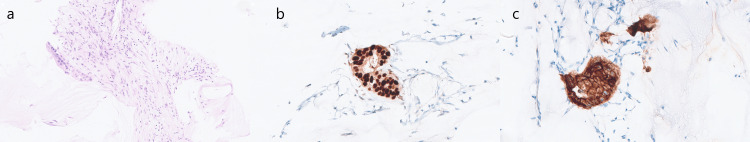
Histopathological analysis of the sinus lesion (a) H&E showing neoplastic glandular structures within fibrous stroma; (b) immunohistochemistry positive for CDX2 with strong nuclear staining, supporting gastrointestinal origin; (c) cytoplasmic CK7 expression, consistent with pancreatobiliary phenotype.

A transnasal endoscopic biopsy was performed on April 1, 2025. Histological analysis showed large mucin lakes interspersed with columnar epithelial cells demonstrating nuclear atypia and prominent nucleoli (Figure 5). Immunohistochemistry revealed diffuse CK7 positivity, CK20 negativity, and strong CDX2 expression, supporting a gastrointestinal origin, most consistent with pancreatic adenocarcinoma [7].

The case was discussed at a multidisciplinary tumor board. Given the presence of multiple peritoneal nodules and confirmed sinonasal metastasis, systemic chemotherapy was resumed. In case of significant local ENT symptoms, radiotherapy to the sinonasal region was proposed as part of the therapeutic strategy.

## Discussion

Sinonasal metastases are rare and often pose significant diagnostic challenges, but their clinicopathological features have been well described in larger series [6]. Ruggeri et al. diagnosed and treated 67 patients with malignant tumors located in the nasal cavity and paranasal sinuses. Eight patients had metastases in the nasal cavity and paranasal sinuses [9]. A review by López et al. identified fewer than 16 reported cases of metastases to the sinonasal tract, most of which originated from renal, breast, or lung cancers [2]. Thus, previous reports have described rare cases of sinonasal metastases from gastrointestinal primaries, including the colon [10,11] and stomach [12]. However, none has involved a pancreatic origin.

To our knowledge, our report is the first documented case of ethmoido-maxillary metastasis from pancreatic adenocarcinoma, reinforcing the importance of histological confirmation in patients with atypical sinus symptoms and a known oncologic history. Metastatic involvement of the paranasal sinuses from pancreatic adenocarcinoma is exceedingly rare, with no other case reports in the literature [3,4].

The mechanism behind metastasis to the sinonasal tract, particularly from abdominal primaries, remains poorly understood. The proposed route is hematogenous, via valveless venous pathways such as Batson’s plexus, which allows retrograde flow from abdominal to cranial structures during episodes of increased intrathoracic or abdominal pressure [4,13]. Given the pancreas’s usual metastatic pattern to the liver, peritoneum, and lungs, this cranial spread is highly atypical. Distinguishing a primary sinonasal tumor from a metastasis of digestive origin relies mainly on histology and immunohistochemistry. In our case, the tumor expressed CDX2 and CK7, but not CK20, a profile more consistent with a pancreatic origin. Colorectal cancers usually express both CDX2 and CK20, but not CK7. Gastric cancers may express all three markers, but often show different patterns and additional markers like MUC2. When combined with the known pancreatic primary and clinical context, these findings strongly support the diagnosis of a pancreatic metastasis to the sinonasal region. Treatment is largely palliative due to the advanced stage at diagnosis. Options include endoscopic surgical debulking for symptom relief, radiotherapy, and systemic therapy tailored to the primary malignancy [2]. In selected cases, aggressive local therapy may be justified for symptom control, especially when orbital or cranial complications are imminent. Prognosis remains poor; median survival is typically measured in months [3]. Nonetheless, timely recognition of such metastases allows for appropriate symptom management and informed decision-making.

## Conclusions

Sinonasal metastasis from pancreatic adenocarcinoma is an extremely rare entity that should be considered in patients with known malignancy and atypical nasal symptoms. Given the overlap with benign sinus disease, prompt imaging and biopsy are essential. Immunohistochemistry plays a critical role in confirming diagnosis. Though prognosis is poor, early identification can guide palliative treatment and improve quality of life. Awareness of such atypical metastatic patterns is vital for timely and accurate clinical management.
